# A random acceleration model of individual animal movement allowing for diffusive, superdiffusive and superballistic regimes

**DOI:** 10.1038/s41598-017-14511-9

**Published:** 2017-10-30

**Authors:** Paulo F. C. Tilles, Sergei V. Petrovskii, Paulo L. Natti

**Affiliations:** 1 Departamento de Matematica, Universidade Federal de Santa Maria, Santa Maria, Brazil; 2 Department of Mathematics, University of Leicester, Leicester, LE1 7RH UK; 3 Departamento de Matematica, Universidade Estadual de Londrina, Londrina, Brazil

## Abstract

Patterns of individual animal movement attracted considerable attention over the last two decades. In particular, question as to whether animal movement is predominantly diffusive or superdiffusive has been a focus of discussion and controversy. We consider this problem using a theory of stochastic motion based on the Langevin equation with non-Wiener stochastic forcing that originates in animal’s response to environmental noise. We show that diffusive and superdiffusive types of motion are inherent parts of the same general movement process that arises as interplay between the force exerted by animals (essentially, by animal’s muscles) and the environmental drag. The movement is superballistic with the mean square displacement growing with time as $\langle {x}^{2}(t)\rangle \sim {t}^{4}$ at the beginning and eventually slowing down to the diffusive spread $\langle {x}^{2}(t)\rangle \sim t$. We show that the duration of the superballistic and superdiffusive stages can be long depending on the properties of the environmental noise and the intensity of drag. Our findings demonstrate theoretically how the movement pattern that includes diffusive and superdiffusive/superballistic motion arises naturally as a result of the interplay between the dissipative properties of the environment and the animal’s biological traits such as the body mass, typical movement velocity and the typical duration of uninterrupted movement.

## Introduction

Dispersal is a common property of all animal and plant species^1,2^. Whilst dispersal of plants occurs predominantly due to the seeds transport by environmental flows (such as, depending on the species traits, wind or water flows), dispersal of animals typically happens due to their self-motion. In this paper, we focus on the dispersal of animals. Understanding dispersal properties is important for many reasons. It is a primary factor affecting the rate and extent of biological invasions^3–5^ and the survival/extinction of species in a fragmented habitat^6–9^, these two processes being mainly responsible for species extinctions and biodiversity loss worldwide^10–12^. Dispersal is also known to control the spread of infectious diseases^13–15^.

Dispersal of a population occurs due to the movement of its individuals. Individual animal movement can be quantified by the probability density function $\rho (x,t)$ (pdf, also known as the dispersal kernel) of the distance *x* traveled over a given time *t*, and/or by the moments of the pdf^16,17^. In particular, the second moment $\langle {x}^{2}(t)\rangle$ called the mean squared displacement (MSD) is used frequently. In case the movement can be described by a stationary stochastic process, the MSD usually depends on time as a power law (but see^18^ for a discussion of singular cases), i.e. $\langle {x}^{2}(t)\rangle \sim {t}^{\alpha }$ where *α* > 0, the cases *α* = 1, *α* < 1 and *α* > 1 corresponding to the ordinary diffusion (Brownian motion), subdiffusion and superdiffusion, respectively^19,20^.

After the groundbreaking report that some bird species perform Levy walks or flights^21^, over the last two decades there has been an intense discussion as to whether animals move predominantly diffusively or superdiffusively^20,22–26^ and how the observed pattern may depend on the properties of the environment^27,28^, on the population structure and the individual differences^29–32^, on the temporal and spatial scales of the observations^33–36^, as well as on the details of the statistical analysis and/or the interpretation of the movement data^37,38^. Many simple, biological, plausible mechanisms for Levy walks (and so for superdiffusion) have been identified, e.g. see^39^ for a review and summary. There is considerable evidence of superdiffusive animal movement available from empirical studies and statistical inferences (see the references above). However, there is relatively little work trying to explain (rather than only describe) the observed movement pattern by developing a relevant theory or a mechanistic model^40–42^. A theory that would relate the movement pattern to animal’s biological traits and to the properties of the environment is required to ensure a good understanding and an efficient control of a variety of ecological phenomena where the dispersal is a key factor. One recent tendency in animal movement studies that bolsters the need for a unifying theory is the growing amount of high resolution movement data when the position of the animal on its movement path is recorded with a very small time interval, almost continuously. That apparently opens a possibility to understand the factors affecting the animal movement on a very fine time scale. One open question is how the interaction between processes occurring at different time scales can affect the movement pattern, in particular to what extent the animal’s movement behavior at a small time scale may affect the movement properties (e.g. the dispersal rate) at a larger time scale. In this paper we do not consider any specific movement data; instead, we endeavor to address the above issues by making some first steps in developing a unifying multiscale theory of animal movement.

In its movement, the animal frequently receive information from the environment through various cues or ‘signals’ which can be visual, auditory, olfactory or tactile origin^24,43,44^. The ability to react to cues through a range of behavioral responses is an essential property of all animals. Animal reacts to the clues by making ‘decisions’ to alter its movement accordingly (cf. “why move?” or “when and where to move?”^44^), i.e. to change its velocity. In particular, when an animal is in the foraging or searching mode, both its speed and movement direction change frequently (usually resulting in a complicated movement path). Change in velocity means a movement with an acceleration and there is considerable empirical evidence showing that foraging animals accelerate/decelerate all the time, e.g. see^45,46^. One can expect that animal’s immediate behavioral response to cues by exerting force (which is a prime physiological reaction to a stimuli^47^) can affect the movement pattern substantially, and indeed it has been shown recently that such response can turn diffusive movement to superdiffusion^48,49^.

In this paper, we consider a general model of individual animal movement described mathematically by the Langevin equation^50–53^ with a non-Wiener stochastic forcing; the latter being a description of the animal’s response to the clues received from the environment. Two of the existing theoretical approaches to stochastic movement such as the velocity jump process and the standard Langevin equation with a Wiener forcing are shown to be the limiting cases of our model. Our model takes into account two main factors affecting any animal movement, i.e. the force exerted by the animal (as is necessary for its self-propelled movement) and the drag or friction from the environment. The relative importance of these factors depends on the properties of the environment and the movement characteristics, e.g. as quantified by the Reynolds number^54^. In case of sufficiently small Reynolds numbers when the drag is described by the Stoke’s law, the equation becomes linear and is analytically tractable. We show that an essential property of the movement is the existence of long term superdiffusive transients. The animal can move superdiffusively over a considerable time before the process eventually converges to the diffusive movement. We calculate the duration of the superdiffusive stage and the value of the diffusion coefficient (which becomes relevant in the large-time limit) and show that they are functions of the ratio of the animal’s body mass and the drag coefficient. By discussing our results in a broader context of animal movement studies, we also show that they can shed a new light on the big question as to how internal and external factors interact to produce the observed movement patterns.

## Model and Method

Consider an animal foraging in a uniform (unstructured) environment. The environment is assumed to be stationary in the statistical sense, i.e. any existing noise or stochasticity that is perceived by the animal through various clues and signals is a stationary stochastic process. We consider the case of continuous, ‘uninterrupted’ animal’s movement (i.e. it may include occasional brief stops but not periods of immobility such as required for rest, feeding, mating etc.). We therefore focus our analysis on a relatively short time scale. On a longer time scale the movement is known to commonly exhibit intermittency where periods of activity (movement) alternate with periods inactivity (rest)^55–57^. As a part of the intermittent movement pattern, relatively long periods of uninterrupted movement (e.g. 10–100 sec for various insect species) are common and they are observed both in natural environments and under controlled laboratory conditions^34,55–57^. Good understanding of the uninterrupted movement is therefore required; once its properties are revealed and analyzed, it can be upscaled to include intermittency^58^.

We assume that the animal’s position at a given time *t* is represented by a single point (e.g. its center of mass), say *x*. Animal movement along its trajectory is conditioned by physical laws and is described by the equation of movement which, considering its projection onto an axis *x*, can be written in the following scalar form:

1 $$m\ddot{x}=\sum _{i\mathrm{=1}}^{n}{F}_{i}.$$

where the dot denotes the derivative with regard to time, *m* is the animal’s mass, and *F*_1_, … *F*_*n*_ are various forces acting on the animal. In this paper, we mostly focus on animals moving within viscous media such as air or water. In this case, the forces acting on the moving animal include the force exerted by the animal itself, drag or friction, gravity and buoyancy. Equation (1) is essentially the Newton’s Second Law in case the forces are deterministic and it turns into the Langevin equation if the forces have a stochastic origin (see below). In case axis *x* is chosen in a horizontal direction, gravity and buoyancy have no effect. Dividing Eq. (1) by *m*, we then arrive at

2 $$\ddot{x}=-F(\dot{x})+a(t),$$

where *a*(*t*) is the force exerted by the animal per its unit mass, which we for convenience call the acceleration, and $F(\dot{x})$ describes the drag (per unit mass) where the minus is added in order to emphasize that the drag is always directed against velocity. Note that, in order to move along, the animal has to exert force most of the time; even in the case of the movement with a constant velocity, i.e. when $\ddot{x}=0$, a force is required to overcome the effect of drag.

In a general case, the function *F* is nonlinear. For small velocities or for small Reynolds numbers (up to $Re\sim {10}^{3}$), the drag is well described by the linear term (the Stokes law), i.e. $F(\dot{x})\approx \gamma \dot{x}$, where parameter *γ* quantifies the dissipative properties of the environment. The linear approximation still provides a reasonable approximation of the drag in the intermediate range of Reynolds numbers between 10^3^ and 10^4^, although the nonlinear corrections (known as Oseen’s approximation) becomes increasingly important^59^. For the sake of analytical tractability, we restrict our analysis to Reynolds numbers being small to intermediate where the linear approximation of the drag is still valid. Relevant animal species include small insects, small birds, bats and small fish^54,60^.

The animal movement as a response to external cues is formally described as a sequence of ‘bouts’. We assume that a bout ends (and the next bout starts) when the animal obtains a cue from the environment. Upon obtaining a cue, the animal reacts by changing the value of the force in order to amend its velocity. The precise dependence of the force on time is likely to be complicated; for the sake of simplicity, here we assume that it is approximately constant between any two subsequent cues. Since the environmental signals are considered to be stochastic, the force exerted by the animal is stochastic as well (for a broader discussion of the “bugbear of randomness” in animal movement see ref.^16^). Therefore, we consider *a* to be a random variable described by a certain probability density function *ϕ*(*a*). Each bout is therefore completely determined by its duration and the acceleration exercised throughout the bout. These two variables are of different origin and hence are considered to be statistically independent.

Since there is empirical evidence that the cues affecting animal movement are distributed in time exponentially^31,57^, we consider the following probability density of bout duration:

3 $$\psi (t)=\omega {e}^{-\omega t}.$$

Within a given bout, i.e. during the time when the acceleration has a given value, the animal movement is described by the deterministic Eq. (2) with a constant *a*. On a longer time-scale that includes at least a few bouts, with the value of acceleration during each bout being chosen randomly according to pdf *ϕ*(*a*), Eq. (2) attains stochastic properties. Given the linear approximation of the drag discussed above, Eq. (2) can be formally written as

4 $$\ddot{x}=-\gamma \dot{x}+a(t),$$

where *a*(*t*) is a stochastic process (force) with certain properties. In case *a*(*t*) is a Wiener process^61^, Eq. (4) is called the Langevin equation. The Langevin equation has previously been suggested as an appropriate model of individual animal movement^40,41^ and cell movement^62^, and was shown to have properties consistent with Levy walk^41^. It is indeed a general model of stochastic movement with friction or drag^51–53^. In a somewhat broader context, depending on the properties of the stochastic forcing (e.g. delta-correlated or exponentially correlated) the Langevin equation belongs to a general class of autoregressive processes of the first or second order^63,64^. The properties of Eq. (4), and hence the properties of the corresponding stochastic motion essentially depends on the properties of the force *a*. In our model, the stochastic force is a non-Wiener process. Firstly, *a*(*t*) is not *δ*-correlated but exponentially correlated:

5 $$\langle a(t)a(t+\tau )\rangle ={\nu }_{a}^{2}{e}^{-\omega \tau },$$

where ${\nu }_{a}^{2}$ is the variance of the acceleration probability distribution (see Section A of the supplementary material for the calculation details). Secondly, and in fact even more importantly, the stochastic process is not continuous. Correspondingly, we refer to Eq. (4) as the Langevin equation with a non-Wiener stochastic process (LEnW). As we will show it below, the LEnW describes movement with different and more general properties; in particular, it includes several well-known movement patterns as special cases.

In order to quantify the movement described by the LEnW (4), we need to be able to calculate the position *x* of the animal at an arbitrary moment *t* that arises after a succession of several bouts where the last bout can still be lasting. Before proceeding to this complicated task, a first natural step is to consider the contribution of a single bout, which can then be generalized to account for an arbitrary number of bouts. In order to avoid confusion, we keep the notation *x* for the animal’s position at an arbitrary moment *t* but use *y* to refer to the position during a given bout.

Consider the animal movement during the first bout which has the duration *t*_1_. Since the acceleration *a*(*t*) is assumed to be constant, *a*(*t*)=*a*_1_, the ordinary differential equation (4) can be easily solved resulting in

6 $$v={v}_{0}{e}^{-\gamma t}+\frac{{a}_{1}}{\gamma }(1-{e}^{-\gamma t}),$$

where $v=\dot{y}$ is the animal’s velocity and *v*_0_ is the initial condition. Equation (6) is valid for 0 ≤ *t* ≤ *t*_1_. Integrating (6), we readily obtain the expression for the coordinate:

7 $$y={y}_{0}+\frac{{v}_{0}}{\gamma }(1-{e}^{-\gamma t})+\frac{{a}_{1}}{{\gamma }^{2}}(\gamma t+{e}^{-\gamma t}-1),$$

where *y*_0_ is the initial position of the animal. Once the first bout is completed (i.e. the animal gets a new clue), the animal velocity *v*_1_ and position *y*_1_ are obtained from (6–7) by setting *t* = *t*_1_. Over the second bout, the same solution (6–7) can be used again, but now with the redefined initial conditions *v*_1_ and *y*_1_, which is valid for ${t}_{1} < t\le {t}_{1}+{t}_{2}$ where *t*_2_ is the duration of the second bout. Having this procedure repeated *k* times, we can obtain the velocity and position of the animal at the end of the *k* ^th^ bout:

8a $${v}_{k}={v}_{k-1}{e}^{-\gamma {t}_{k}}+\frac{{a}_{k}}{\gamma }(1-{e}^{-\gamma {t}_{k}}),$$

8b $${y}_{k}={y}_{k-1}+\frac{{v}_{k-1}}{\gamma }(1-{e}^{-\gamma {t}_{k}})+\frac{{a}_{k}}{{\gamma }^{2}}(\gamma {t}_{k}+{e}^{-\gamma {t}_{k}}-1),$$

where *a*_*k*_ and *t*_*k*_ are the acceleration during the *k* ^th^ bout and the duration of the bout, respectively.

The solution to the recurrence relations with initial conditions at rest (*y*_0_ = *v*_0_ = 0) depends on only the accelerations **a**_*k*_ = (*a*_1_, …, *a*_*k*_) and the bout durations **t**_*k*_ = (*t*_1_, …, *t*_*k*_). It does not require much effort to show that the solution can be written as

9 $${y}_{k}=\sum _{n\mathrm{=1}}^{k}\frac{{a}_{n}}{{\gamma }^{2}}[\gamma {t}_{n}+({e}^{-\gamma {t}_{n}}-1)\exp (-\gamma \sum _{j=n+1}^{k}{t}_{j})],$$

but equation (9) still needs to be linked to the animal position at a given time *t*. If we realize that *t* is a continuous variable that should be located inside the period of any of the infinitely many bouts that may compose the movement, then whenever we are computing the contribution of a given sequence of *k* bouts, we may always define a variable *x*_*k*_ that corresponds to the spatial position of the animal at a given time *t* that lies within *k* ^*th*^ bout, thus satisfying ${\sum }_{n=1}^{k-1}{t}_{n} < t < {\sum }_{n=1}^{k}{t}_{n}$. Correspondingly, the last bout duration *t*_*k*_ in equation (9) is defined as follows:

10 $${t}_{k}=t-\sum _{n\mathrm{=1}}^{k-1}{t}_{n},$$

and as a result we may effectively truncate the sequence of bouts to obtain a position *x*_*k*_ at any given time *t* that we want, as long as it satisfies the condition $t > {\sum }_{n=1}^{k-1}{t}_{n}$. Thus, we formally obtain the conditioned equation

11 $${x}_{k}=\sum _{n=1}^{{k}^{\ast }}\frac{{a}_{n}}{{\gamma }^{2}}[\gamma {t}_{n}+({e}^{-\gamma {t}_{n}}-1)\exp (-\gamma \sum _{j=n+1}^{{k}^{\ast }}{t}_{j})],$$

which gives us the spatial position *x*_*k*_ at a time *t*, as a contribution from a sequence of *k* bouts. The upper index notation *k* ^*^ is used to indicate that constraint (10) should be used in order to make the equation consistent.

## Analysis and Results

We first have to decide about the properties of an appropriate probability density function *ϕ*(*a*). Recall that we consider movement in a homogeneous and isotropic environment; the absence of a preferred direction is ensured by the requirement that the average value of the acceleration over the ensemble of all bouts is zero, $\langle a\rangle =0$. It is also reasonable to assume that the average energy expenditure is finite; correspondingly, the variance ${\nu }_{a}^{2}$ of the acceleration probability distribution *ϕ*(*a*) should also be finite. In its turn, it means that *ϕ*(*a*) must be either a function with finite support or a function that decays sufficiently fast at large $|a|$. As a starting point, we therefore consider the case where acceleration is described by a Gaussian probability distribution, i.e.,

12 $$\varphi (a)=\frac{{e}^{-\frac{{a}^{2}}{2{\sigma }^{2}}}}{\sqrt{2\pi {\sigma }^{2}}}.$$

This specific choice of *ϕ*(*a*) will enable us to obtain a formal equation for the dispersal kernel *ρ*(*x*, *t*) (see part B of the supplementary material) and to analyze the properties of both the tail of the distribution and its spreading rate.

**Figure 1:**
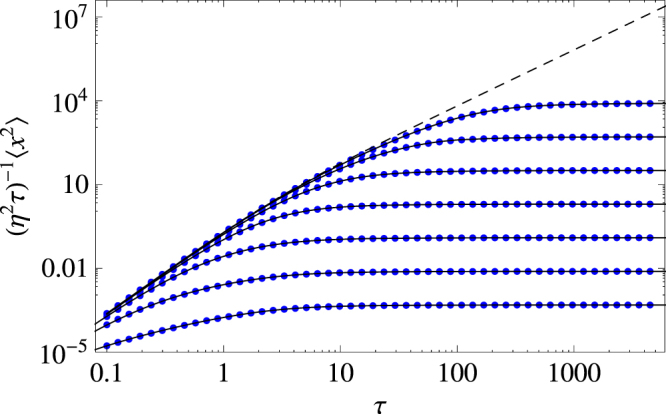
Rescaled MSD for the Gaussian dispersal process. Dotted (blue) curves are obtained from ${10}^{7}$ samples of Monte Carlo simulations, while continuous (black) curves were determined by Eq. (13). From top to bottom, $\zeta ={4}^{n}$, with $n=-\mathrm{3,}\,-\mathrm{2,}\,-1,\,0,\,1,\,2,\,3$. The dashed curve represents the zero drag regime of the MSD $\langle {z}^{2}(\tau )\rangle$, as defined in equation (18), which describes the initial spread (small asymptotics) for the animal, regardless of the value of $\zeta$.

The quantities that characterized the animal’s movement, i.e. the dispersal kernel and the MSD, are obtained by integrating over all possible bout sequences and duration. After tedious analytical calculations (see parts B and C of the supplementary material for details), we obtain the following exact expression for the MSD:

13 $$\langle {x}^{2}(\zeta ,\tau )\rangle =\frac{2{\eta }^{2}}{\zeta }\{\frac{\tau }{\zeta }-\frac{1+2{(1+\zeta )}^{2}}{2{\zeta }^{2}(1+\zeta )}+\frac{1}{\zeta -1}[\frac{{e}^{-2\zeta \tau }}{2{\zeta }^{2}}+{e}^{-\zeta \tau }(\frac{\zeta -2}{{\zeta }^{2}}-\frac{{e}^{-\tau }}{1+\zeta })+{e}^{-\tau }]\},$$

where the rescaled (dimensionless) variable and parameters

14 $$\tau =\omega t,\quad \eta =\frac{\sigma }{{\omega }^{2}},\quad \zeta =\frac{\gamma }{\omega },$$

were used in order to reduce the dimensionality of the parameter space. Even though we have explicitly assumed that condition $\gamma  > \omega$ ($\zeta  > 1$) was satisfied, the expression for the MSD appears to be valid for all values of parameters because it matches perfectly every simulation data that we have used as a comparison; see Fig. 1.

**Figure 2:**
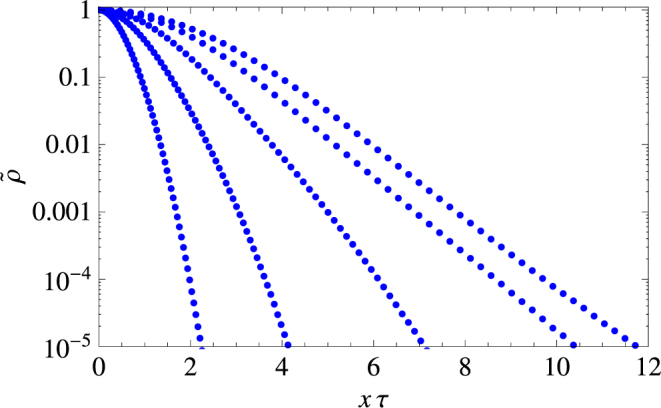
Renormalized probability distribution function $\tilde{\rho }(x,\tau )=\rho {(0,\tau )}^{-1}\rho (x,\tau )$ shown at different moments of the rescaled time corresponding to an early stage of animal movement, left to right: $\tau =1,\,2,\,4,\,8,\,16$. The data (as shown by blue dots) are obtained using 10^9^ Monte Carlo simulations. Note that the pdf has an exponential tail.

**Figure 3:**
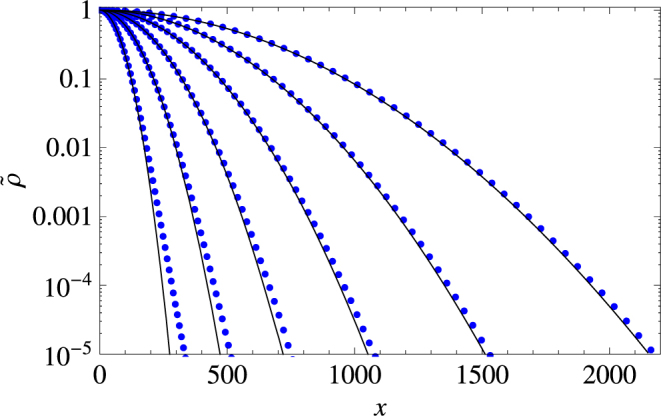
Renormalized probability distribution function $\tilde{\rho }(x,\tau )=\rho {(0,\tau )}^{-1}\rho (x,\tau )$ shown at large time, left to right: $\tau =\mathrm{32,\; 64,\; 128,\; 256,\; 512,\; 1024}$. The data (shown by blue dots) are obtained using 10^9^ Monte Carlo simulations. Black curves show the the Gaussian dispersal kernel (17) that arises in the large time limit.

In the small-time limit $\tau \ll 1$, Eq. (13) turns into

15 $$\langle {x}^{2}(\tau )\rangle \sim \frac{{\eta }^{2}}{4}{\tau }^{4},$$

and hence obviously predicts a super-ballistic spread. In the large-time limit $\tau \gg 1$, i.e. when we can neglect all the exponentials, Eq. (13) turns into the following:

16 $$\langle {x}^{2}(\zeta ,\tau )\rangle \simeq \frac{2{\eta }^{2}}{{\zeta }^{2}}\tau -\frac{{\eta }^{2}[1+2{(1+\zeta )}^{2}]}{{\zeta }^{3}(1+\zeta )}\equiv {{\rm{\Delta }}}_{d}^{2}(\zeta ,\tau ),$$

thus predicting the diffusive spread in the large-time asymptotics with the diffusion coefficient given as $D={\eta }^{2}/{\zeta }^{2}={\sigma }^{2}/({\gamma }^{2}\omega )$. Slowing down from the superballistic spread at small time to the diffusive spread at large time is consistent with the changes in the shape of the probability distribution function, see Figs 2 and 3. The superdiffusive spread accounts for a fatter tailed, exponential type of curve (see the top panel) in the small time regime, the duration of which is on the order of a several bouts. Asymptotically the pdf tends to a regular Gaussian type of curve, in the same way as observed in ref.^49^, thus enabling us to make a very accurate guess for the pdf, i.e.,

17 $$\rho (x,\zeta ,\tau )\simeq \frac{1}{\sqrt{2\pi {{\rm{\Delta }}}_{d}^{2}(\zeta ,\tau )}}{e}^{-\frac{{x}^{2}}{2{{\rm{\Delta }}}_{d}^{2}(\zeta ,\tau )}},$$

which agrees with simulations almost perfectly.

The presence of the fast-spreading transient dynamics may be best appreciated when we consider the MSD for small values of *γ*: the smaller the friction/dragging parameter *γ*, the longer the system remains in the superdiffusive motion. Consider the spread in the limiting case $\zeta =0$:

18 $$\langle {z}^{2}(\tau )\rangle \equiv \mathop{\mathrm{lim}}\limits_{\zeta \to 0}\langle {x}^{2}(\zeta ,\tau )\rangle =\frac{{\eta }^{2}}{3}[{\tau }^{2}(2\tau -3)+6-6(1+\tau ){e}^{-\tau }].$$

It is readily seen (cf. the dashed line in Fig. 1) that, for sufficiently small $\tau$, this limiting case provides a good approximation for the MSD in case of *ζ* > 0. Therefore, regardless of the specific value of *ζ*, during the initial stage of spread the animal always moves superballistically. This agrees well with an earlier result^48^ that super-ballistic spread with $\langle {x}^{2}\rangle \sim {\tau }^{3}$ may be a plausible scenario in a frictionless environment.

Now we may go even further and estimate how long an animal performs the super-diffusive spread described by Eq. (18), i.e., we may obtain the typical duration of this type of movement via a confidence parameter ${\varepsilon }_{sd}\in [0,1]$, as defined by the following relation:

19 $$\frac{\langle {x}^{2}(\zeta ,{\tau }_{sd})\rangle }{\langle {z}^{2}({\tau }_{sd})\rangle }\equiv {\varepsilon }_{sd},$$

which is a measure of proximity between the two functions: the closer it is to unity, the more similar the two functional forms are. If expand the left side of Eq. (19) in the region where both *τ* and *ζ* are very small, and take only the leading order terms, then it is possible to obtain an approximate relation for the time *τ*_*sd*_ at which the proximity becomes smaller than the confidence parameter *ε*_*sd*_,

20 $${\tau }_{sd}(\zeta ,{\varepsilon }_{sd})\approx \frac{3(1-{\varepsilon }_{sd})}{2\zeta }.$$

**Figure 4:**
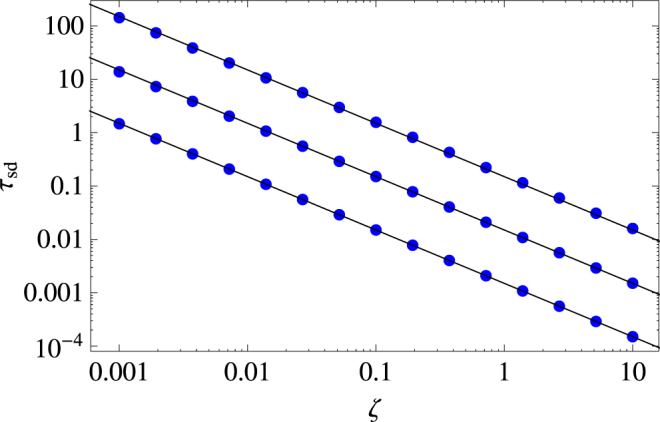
Duration ${\tau }_{sd}$ of superdiffusive spread as a function of $\zeta =\gamma /\omega$ obtained for different values of the confidence parameter ${\varepsilon }_{sd}$ which quantifies the proximity between the actual transient regime of spread and its superdiffusive asymptotics. From top to bottom, ${\varepsilon }_{sd}=0.9,\,0.99,\,0.999$. Dots represents the numerical evaluation of Eq. (19), the solid lines show the approximate relation given by Eq. (20).

Figure 4 shows the numerical evaluation of the exact relation (19) and the very good approximate description obtained by Eq. (20).

In the same manner, we can also look at the asymptotic times at which the system has nearly converged to the diffusive spread. For this, we are going to define another confidence parameter ${\varepsilon }_{d}\in [0,1]$ as

21 $$\frac{\langle {x}^{2}(\zeta ,{\tau }_{d})\rangle }{{{\rm{\Delta }}}_{d}^{2}(\zeta ,{\tau }_{d})}\equiv {\varepsilon }_{d},$$

and the closer to unity this value gets, the better is the spreading approximated by the asymptotic diffusion. In order to obtain an approximate relation for *τ*_*d*_, we should look at the left side of Eq. (21) in the limit of sufficiently large *τ*, where the ratio may be approximated by

22 $$\frac{\langle {x}^{2}(\zeta ,\tau )\rangle }{{{\rm{\Delta }}}_{d}^{2}(\zeta ,\tau )}\approx 1-\frac{(1+\zeta )[{e}^{-2\zeta \tau }+2{\zeta }^{2}{e}^{-\tau }-2(2-\zeta ){e}^{-\zeta \tau }]-2{\zeta }^{2}{e}^{-(1+\zeta )\tau }}{3+\zeta [1-2\zeta -2{\zeta }^{2}-2(1-{\zeta }^{2})\tau ]}\simeq 1+\frac{2(1+\zeta )(2-\zeta ){e}^{-\zeta \tau }}{3+\zeta [1-2\zeta -2{\zeta }^{2}-2(1-{\zeta }^{2})\tau ]}.$$

With this expression we obtain a complicated solution in terms of *τ*_*d*_, which may be simplified if we look only at the leading order term in the small *ζ* regime, thus leading to

23 $${\tau }_{d}(\zeta ,{\varepsilon }_{d})\approx \frac{1}{\zeta }\{\frac{3}{2}+W[\frac{2}{{e}^{\mathrm{3/2}}(1-{\varepsilon }_{d})}]\},$$

where $W(\bullet )$ is the Lambert W function, as defined by the inverse relation of

24 $$x=W(x){e}^{W(x)}.$$

**Figure 5:**
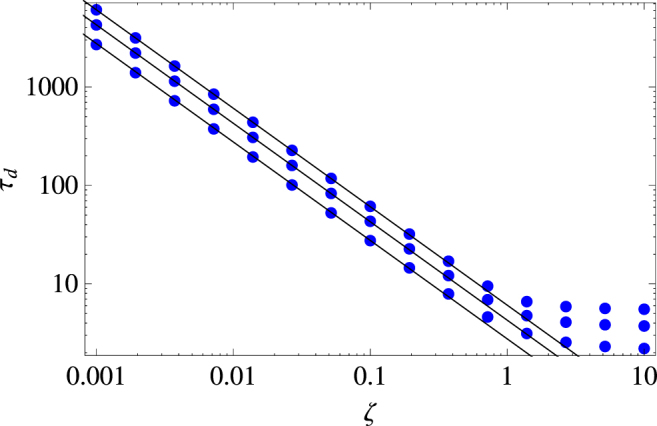
Time ${\tau }_{d}$ required for the animal’s transient movement process to converge to the ordinary diffusive spread, with the proximity between the two regimes beng quantified by the confidence parameter ${\varepsilon }_{d}$. From top to bottom, ${\varepsilon }_{d}=0.999,\,0.99,\,0.9$. Dots represents the numerical evaluation of Eq. (21), the solid lines show the approximate relation given by Eq. (23).

Figure 5 shows the numerical evaluation of the exact relation (21) and the very good approximate description for small *ζ* obtained by Eq. (23).

The results above are obtained under the assumption that the pdf of acceleration values is given by a normal distribution, cf. Eq. (12). In the next section, however, we will show that the expression (14) for the MSD remains valid in a much more general case, in fact for any *ϕ*(*a*) with zero mean and a finite variance.

## Discussion of Limiting Cases and Multiple Scales

A closer look at the expression (13) for the MSD readily reveals the existence of a few different time scales. In the previous section, we have shown that $\langle {x}^{2}\rangle \sim {\tau }^{4}$ when *τ* is sufficiently small. However, it appears possible to make a more detailed analysis based on the existence of the two time scales of the problem as given by 1/*γ* and 1/*ω*.

Let us consider first the low drag limit, $\zeta =\gamma /\omega \ll 1$, i.e. the parameter range where long transients are expected to be prominent. We notice that in this range *e* ^−*τ*^ decays much faster than *e* ^−*ζτ*^, so that there is a movement stage where the former exponent can be neglected yet the latter one should be kept, i.e. $\tau \gg 1$ but the product (*ζτ*) is not necessarily large. Correspondingly, Eq. (13) can be approximated as

25 $${\langle {x}^{2}\rangle }_{\zeta \ll 1}\approx \frac{2{\eta }^{2}}{{\zeta }^{3}}(\zeta \tau -\frac{3}{2}+2{e}^{-\zeta \tau }-\frac{1}{2}{e}^{-2\zeta \tau })=\frac{2{\sigma }^{2}}{{\gamma }^{3}\omega }[\gamma t-(1-{e}^{-\gamma t})-\frac{1}{2}{(1-{e}^{-\gamma t})}^{2}].$$

The right-hand side of Eq. (25) is immediately recognized (e.g. see page 35 in^65^) as the MSD of the standard Langevin equation with a Wiener process, the variance *q* ^2^ of the process being given by ${q}^{2}=2{\sigma }^{2}/\omega$. By considering its small-time asymptotics ($\gamma t\ll 1$), it is readily seen that the movement at the early stages is superballistic, i.e.,

26 $${\langle {x}^{2}\rangle }_{\zeta \ll 1}\sim \frac{2{\sigma }^{2}}{3\omega }{t}^{3}.$$

At the first sight, this result may lead to a confusion as the Langevin equation is usually associated to an initial ballistic spread with $\langle {x}^{2}\rangle \sim {t}^{2}$. However, here we recall that the prediction of ballistic spread is only valid under the assumption that the initial animal velocity is ${v}_{0}^{2}=q/(2\gamma )$, which means that the animal is in a ‘thermal equilibrium’ with its (stationary) environment, i.e. it has had a sufficient time to adapt to it; see refs^49,65^ for details. On the contrary, in our model the animal is at rest (${v}_{0}=0$) when the movement starts. We also mention here that the small-time superballistic limit of the Brownian motion has recently been experimentally observed in a physical system^66^.

The Brownian motion arising in the low-drag limit can be further justified if we look at Eq. (5) in the limit where $\omega \to \infty$, as the acceleration autocorrelation function becomes negligibly small everywhere except for $\tau =0$, thus resembling the delta correlation of the Wiener process. However, this comparison must be taken with caution, as the stochastic acceleration assumed here cannot be taken as a noise term on a Langevin equation because it is not a continuous process and it does not necessarily present Gaussian increments, as both conditions are required in order to describe a Wiener process.

Let us now consider the opposite limit of a high drag, $\zeta \gg 1$. Then ${e}^{-\tau }$ decays much slower than ${e}^{-\zeta \tau }$, so that there is a movement stage where the former exponent should be kept but the latter one can be neglected, i.e. $\zeta \tau \gg 1$ where *τ* is not necessarily large. In this high drag limit, equation (13) can be approximated by

27 $${\langle {x}^{2}\rangle }_{\zeta \gg 1}\approx \frac{2{\eta }^{2}}{{\zeta }^{2}}(\tau -1+{e}^{-\tau })=\frac{2{\sigma }^{2}}{{\gamma }^{2}{\omega }^{2}}(\omega t-1+{e}^{-\omega t}),$$

which coincides with the MSD predicted by the velocity jump process^67,68^. In particular, it is readily seen that the small-time asymptotics ($\omega t\ll 1$) of Eq. (27) predicts a ballistic spread:

28 $${\langle {x}^{2}\rangle }_{\zeta \gg 1}\sim \frac{{\sigma }^{2}}{{\gamma }^{2}}{t}^{2}.$$

In order to further demonstrate the equivalence between the two models, let us recall that the animal’s velocity in the *k* th bout (i.e. during the time interval between two consequent signals) is given by the following expression:

29 $$v(t)={v}_{k}{e}^{-\gamma (t-{t}_{k})}+\frac{a}{\gamma }[1-{e}^{-\gamma (t-{t}_{k})}],\quad {t}_{k}\le t\le {t}_{k+1}={t}_{k}+\delta ,$$

cf. Eq. (6), where ${v}_{k}=v({t}_{k})$ and *δ* is the duration of the given interval. It is readily seen that $v(t)$ converges in the large time limit, $v(t)\to {v}_{f}=a/\gamma$. However, the ‘large time limit’ is only achievable if there is sufficient time for this, i.e. *δ* is sufficiently large. On the other hand, the rate of convergence is obviously given by *γ*. Therefore, the ‘final’ velocity ${v}_{f}$ is reached sufficiently fast (and then can be considered as the constant velocity of the $k$ th bout) if the time required for $v(t)$ to effectively reach ${v}_{f}$, i.e. $\mathrm{1/}\gamma$, is much shorter than the typical duration of the bout, i.e. $\mathrm{1/}\omega$. This is exactly what is given by the high drag limit $\zeta =\gamma /\omega \gg 1$. The larger *ζ* is, the more closely our model approximates the velocity jump process.

We therefore have shown that the movement process described by our model has a few different time scales and, correspondingly, different movement stages. At a very early stage, i.e. as defined by $\tau \ll 1$ and $\zeta \tau \ll 1$ or $t\ll \,{\rm{\min }}\,\mathrm{(1/}\omega ,\mathrm{1/}\gamma )$, the movement starts as a very fast, superballistic spread with $\langle {x}^{2}\rangle \sim {t}^{4}$; see Eq. (15). In the course of time, it slows down to $\langle {x}^{2}\rangle \sim {t}^{3}$ and then eventually to $\langle {x}^{2}\rangle \sim {t}^{2}$; however, which of these stages is dominating depends on the relative drag intensity, i.e. on *ζ*. At large times, i.e. for $\tau \gg 1$ and $\zeta \tau \gg 1$ or $t\gg \,{\rm{\max }}\,\mathrm{(1/}\omega ,\mathrm{1/}\gamma )$, the movement slows down to the diffusive spread, $\langle {x}^{2}\rangle \sim t$.

We now observe that, since the asymptotic diffusive movement is solely defined in terms of the MSD, it is possible to extend our approach onto a more general functional form for the acceleration pdf $\varphi (a)$. The dependence of the MSD in equation (13) on the variables $\tau$ and $\zeta$ appears to be the same for any pdf with zero mean ($\langle a\rangle =0$) and finite variance ($\langle {a}^{2}\rangle ={\nu }_{a}^{2}$), see part D of the supplementary material for details, which means that this more general case is attainable simply by making the parameter change ${\sigma }^{2}\to {\nu }_{a}^{2}$. Now, if we compare the asymptotic limit (16) to the 1D diffusion spread $\langle {x}^{2}\rangle =2Dt$, while taking into account equation (14), we immediately obtain the expression for the diffusion coefficient that quantifies the spread in the large-time limit as

30 $$D=\frac{{\nu }_{a}^{2}}{{\gamma }^{2}\omega }.$$

## Further Discussion and Concluding Remarks

Dispersal is a key processes in ecological dynamics^1,2^. Individual animal movement is one of the dispersal’s key elements^16,17,44^; hence, its good understanding is required to provide a rigorous scientific basis for the management and control of many ecological processes and phenomena. In its turn, such a progress is unthinkable without a comprehensive theory that could explain (rather than only describe) the animal movement patterns by relating them to animal’s movement behavior and the properties of the environment, to eventually describe the animal movement across all relevant spatial and temporal scales^24,35,39,69^.

In this paper, we have endeavored to make some first steps in this direction. In doing so, we started from the first principles, i.e. by considering a general equation of animal movement subject to typical forces acting on the moving body; see Eqs (1 and 2) and the lines between them. Arguably, the most typical forces are the drag from the environment and the force exerted by the animal required to overcome the drag. By assuming additionally that the animal reacts to random cues or signals received from the environment, and that the movement occurs with small or intermediate Reynolds numbers, our model becomes a Langevin equation with a non-Wiener discontinuous stochastic process. Here we emphasize that, although the idea to use the Langevin equation to describe biological movement is by no means new^19,40,41,53^, usually the stochastic process (forcing) associated with the movement is assumed to be of Wiener type. In contrast, we considered a non-Wiener process which has biologically more relevant properties, at least in the context of the individual animal movement. We showed that it results in a more general model, which includes the standard Langevin equation (i.e. with Wiener noise) and the velocity jump model as its limiting cases.

Using this rather general model of stochastic motion, we showed that, when started from rest, the emerging movement will always be superballistic at its early stage with $\langle {x}^{2}(t)\rangle \sim {t}^{4}$. The duration of this stage may however be very short; whether the early stage of movement is effectively represented by $\langle {x}^{2}(t)\rangle \sim {t}^{4}$ or $\langle {x}^{2}(t)\rangle \sim {t}^{3}$ or $\langle {x}^{2}(t)\rangle \sim {t}^{2}$ will depend on the relative intensity of the drag and the animal’s self-forcing (‘acceleration’) in response to the cues; see Table 1. Eventually, the movement will slow down to converge in the large-time limit to a normal diffusion (Brownian motion). As follows from Eq. (23), the duration ${\tau }_{d}$ of the superdiffusive movement is proportionate to the frequency of the clues and inverse proportionate to the strength of the dissipation force (friction or drag), ${\tau }_{d}\sim \omega /\gamma$.

**Table 1:** Effective small-time asymptotics of the animal movement as given by the dependence of the MSD on time for different relative intensity of the drag quantified by the ratio $\zeta =\gamma /\omega$; see Section “Discussion of Limiting Cases” for details.

Drag intensity	low-drag limit, $\zeta \ll 1$	intermediate drag, $\zeta \sim 1$	high-drag limit, $\zeta \gg 1$
$\langle {x}^{2}(t)\rangle$	$\sim {t}^{3}$	$\sim {t}^{4}$	$\sim {t}^{2}$

We now recall that in our model (4) the balance of forces is considered per unit mass. Having compared it with the baseline Eq. (1), it is readily seen that $\gamma =c/m$ where $m$ is the mass of the animal and $c$ is the drag coefficient that quantifies the effect of drag on the moving body as a whole (hence depending on both the dissipative properties of the environment and on the shape of the body). For the duration of the superdiffusive stage we therefore obtain ${\tau }_{d}\sim m\omega /c$, and for the diffusion coefficient $D={m}^{2}{\nu }_{a}^{2}/({c}^{2}\omega )$. These expressions therefore explicitly demonstrate how the properties of the movement arise from the interplay between the biological traits (e.g. as quantified by $m$ and ${\nu }_{a}^{2}$) and the properties of the environment (as quantified by *c* and *ω*).

In our analysis, technically, we focused on a 1D movement. However, it is readily seen that, at least in the case where the pdf of the animal’s force is described by a normal distribution, a 2D case splits into a direct product of the two 1D cases (as given by the movement in the horizontal directions *x* and *y*); see^48^ for details. Since the environment is assumed to be homogeneous and isotropic, $\langle {{\bf{r}}}^{2}\rangle =\langle {x}^{2}\rangle +\langle {y}^{2}\rangle =2\langle {x}^{2}\rangle$, which implies that the expression (14) for the MSD remains valid in the 2D case subject to the extra numerical coefficient. An extension of our results onto a 3D case is less straightforward as the inclusion of the third (vertical) dimension would require to account for the effects of gravity and buoyancy.

Once the movement has started, it will be superdiffusive at the first stage of the dispersal. Note that in our analysis we assumed that the animal performs an uninterrupted foraging movement, i.e. without stops such as may be required for rest. An immediate application of our results is therefore limited to the corresponding time scale. On a longer time scale, periods of uninterrupted movement would necessarily alternate with periods of rest. The duration of both periods of movement and periods of rest can be regarded as a random variable described by certain probability distributions^19,70^. Whilst the distribution of movement time is known to be close to exponential^31,57^, the distribution of rest time can be exponential for some species but can exhibit fat tails for others^57^. In the former case, the short-term movement can be upscaled to include the periods of rest^58^. In this case, the general result is that the effect of rests leads to rescaling of the parameter of the corresponding probability distribution but does not change the movement pattern. Hence, if the movement is superdiffusive on the time scale of uninterrupted movement, it will remain superdiffusive on a longer time scale that includes periods of movement and rest^58^. An ecologically relevant time scale of the individual animal movement is given by a typical duration of uninterrupted motion. Correspondingly, one immediate prediction of our model is that whether the movement pattern is diffusive or superdiffusive is to a large extent determined by the ratio of the duration of the superdiffusive stage, e.g. as given by ${\tau }_{d}$, and the typical duration of uninterrupted movement, say $T$. In case ${\tau }_{d}/T\ll 1$, the observed pattern is likely to be diffusive, in the opposite limit ${\tau }_{d}/T\gg 1$ it is likely to be superdiffusive.

In case of longer uninterrupted foraging movement, which is sometimes observed in insects, some avian species and marine species, our results predict that the movement data collected over sufficiently long time will necessarily include both diffusive and superdiffusive bouts. Identification of the movement pattern (e.g. diffusive or superdiffusive) from movement data may then become relative rather than absolute as it will then depend on the observation time, i.e. the period over which the data on animal spatial displacement is collected. In case the observation time is of the same magnitude that the duration of the superdiffusive stage (or shorter), then the pool of data would predominantly consist of bouts corresponding to the superdiffusive movement, so that any relevant analysis would lead to the conclusion of a superdiffusive spread. However, in case the observation time is much longer than the superdiffusive stage, the majority of data will have diffusive properties and hence a conclusion of diffusive movement is likely to be made.

Note that, although it is not our aim here to make a direct quantitative comparison of our theory to specific movement data, there is a general correspondence between our findings and the existing observations on movement of animals in different environments. Indeed, our theory predicts that the animal movement would be predominantly superdiffusive in a low-friction environment but should be predominantly diffusive in a high-friction environment. One example of a high-friction environment is soil. Interestingly, there is no evidence for a superdiffusive movement for soil-dwelling animals, their movement being well described by the ordinary diffusion^29^. This is in a good agreement with our theory: for the movement in a high-friction environment (e.g. soil), the duration of the superdiffusive stage will be negligibly short. On the other hand, a paradigm for animal movement following Levy walks is the flight of birds (in particular, albatrosses^21^). However, the air is a low-friction environment, hence the transient superdiffusive stage of a considerable duration is expected and is likely to dominate in observation data.

## Electronic supplementary material

Supplementary Material
