# Supplementary material for: A random acceleration model of individual animal movement allowing for diffusive, superdiffusive and superballistic regimes
Source: Sci Rep. 2017 Oct 30;7:14364. doi: 10.1038/s41598-017-14511-9 (PMC5662607; doi:10.1038/s41598-017-14511-9)
Supplement: Supplementary file 1 — Supplementary Material [file 41598_2017_14511_MOESM1_ESM.pdf]

Scientific Reports - Supplementary Material

A random acceleration model of individual animal movement allowing for diffusive, superdiffusive and superballistic regimes

Paulo F. C. Tilles, Sergei V. Petrovskii and Paulo L. Natti

A Acceleration autocorrelation function

The process $a(t)$ is a piecewise constant stochastic force defined in terms of two random variables: a sequence of random intensities originated from a probability distribution function (pdf) with zero mean and finite variance, and exponentially distributed waiting times controlling the rate at which the intensity is renewed. It follows from its definition that the autocorrelation function may be written as

$$\langle a(t)\, a(t+\tau)\rangle = \nu_a^2 \varphi(\omega, \tau), \tag{A.1}$$

where ν_a^2 is the variance of the acceleration pdf $\phi(a)$, and $\varphi(\omega, \tau)$ is the probability that both t and $t+\tau$ belong to the same bout. If we denote by $\varphi_k(\omega, t, \tau)$ the probability that both t and $t+\tau$ belongs to the kth bout, then we may write

$$\varphi(\omega, \tau) = \sum_{k=1}^{\infty} \varphi_k(\omega, t, \tau) \tag{A.2}$$

(note that while φ_k depends on the time of reference t, the whole probability φ does not). The $k = 1$ contribution is straightforward to compute, as it is just the probability that the first bout is longer than $t+\tau$, which gives $\varphi_1 = e^{-\omega(t+\tau)}$. But for $k > 1$ we need to compute the joint probability that the total duration of the first $k-1$ bouts is no longer than t and that the kth bout encompasses both t and $t+\tau$. Since the bout durations are exponentially distributed according to equation (3) in the main text, we may use the Gamma distribution

$$\psi_k(t) = \frac{\omega^k t^{k-1}}{\Gamma(k)} e^{-\omega t} \tag{A.3}$$

to account for the pdf of the total duration of the first $k-1$ bouts, which enables us to write the contributions in equation (A.2) as

$$\varphi_k(\omega, t, \tau) = \int_0^t dt' \psi_{k-1}(t') \int_{t+\tau-t'}^{\infty} dt'' \omega e^{-\omega t''} = \frac{(\omega t)^{k-1}}{\Gamma(k)} e^{-\omega(t+\tau)}, \qquad k > 1. \tag{A.4}$$

The sum of all bout contributions gives

$$\varphi(\omega, \tau) = e^{-\omega(t+\tau)} + \sum_{k=2}^{\infty} \varphi_k(\omega, t, \tau) = e^{-\omega\tau}. \tag{A.5}$$

If we insert the result (A.5) back in equation (A.1), it is readily seen that after dividing it by $\langle a^2(t)\rangle = \nu_a^2$, one obtains the normalized autocorrelation function shown in equation (5) of the main text.

B Formal determination of the probability density function

The starting point is the general expression for the spatial position x_k at any given time t, which is already described by Eqs. (11) in the main text. Once x_k is a linear combination of the normally distributed random variables $a_1, \ldots, a_k$, then the probability density for the variable x_k is therefore normally distributed too, i.e.,

$$\rho_k\left(x, t|\mathbf{t}_{k-1}\right) = \frac{\exp\left[-\frac{x^2}{2\Delta_k^2(t,\mathbf{t}_{k-1})}\right]}{\sqrt{2\pi\Delta_k^2\left(t, \mathbf{t}_{k-1}\right)}}, \tag{B.1}$$

where

$$\Delta_k^2\left(t, \mathbf{t}_{k-1}\right) = \frac{\sigma^2}{\gamma^4}\sum_{n=1}^{k^*}\left[\gamma t_n + \left(e^{-\gamma t_n} - 1\right)\exp\left(-\gamma\sum_{j=n+1}^{k^*} t_j\right)\right]^2. \tag{B.2}$$

Note that the probability distribution (B.1) is in fact a *conditioned* probability distribution, i.e. it is only valid for a specific sequence of bouts (as given by their periods $\mathbf{t}_{k-1}$) that took place before the ongoing k^{th} bout. Unconditioned measures, i.e. valid for *any* sequence of bouts, are obtained after we average the conditioned dispersal kernel $\rho_k\left(x, t|\mathbf{t}_{k-1}\right)$ over all possible bout durations for a fixed value of k. In this sense we must consider how all these different configurations of bout durations effectively contribute to the dispersal function in Eq. (B.6). Since the contribution of a given configuration should be weighted by the probability of it actually happening, the unconditioned contribution of a given sequence of k bouts should be obtained from

$$\rho_k\left(x, t\right) = \int \mathcal{D}t_{k-1}\rho_k\left(x, t|\mathbf{t}_{k-1}\right)\int_{t-\sum_{n=1}^{k-1} t_n}^{\infty} dt_k \prod_{j=1}^{k}\psi\left(t_j\right), \tag{B.3}$$

where we have defined a compact notation for 'summing' the contributions of all bout periods up to t_{k-1},

$$\int \mathcal{D}t_{k-1} \equiv \int_0^t dt_1 \int_0^{t-t_1} dt_2 \cdots \int_0^{t-\sum_{n=1}^{k-2} t_n} dt_{k-1}, \tag{B.4}$$

and the last bout period t_k should be superiorly unbounded because the time t is always located within its limits. The variance (B.2) does not depend on t_k, so the last integral may be performed directly, resulting in the expression

$$\rho_k\left(x, t\right) = \omega^{k-1}e^{-\omega t}\int \mathcal{D}t_{k-1}\rho_k\left(x, t|\mathbf{t}_{k-1}\right). \tag{B.5}$$

Hence, as a result of the composite of the animal's random walk, the dispersal kernel is a sum that accounts for all possible bout sequences. Indeed, strictly speaking, by the given time t any number of bouts could have taken place. We therefore write the dispersal kernel as

$$\rho\left(x, t\right) = \sum_{k=1}^{\infty}\rho_k\left(x, t\right), \tag{B.6}$$

where $\rho_k\left(x, t\right)$ is the contribution of a sequence of k bouts. It is important to note that, although exact, the formal expressions (B.5) and (B.6) are not of much practical use, because it is hardly possible to explicitly calculate all the integrals. One way to proceed is to focus on the moments of the dispersal kernel rather than on the kernel itself: as it happens, the calculations become simpler if the integration over space is included.

C Exact expression for the mean squared displacement

Consider the definition of the moment of the order $2n$:

$$\langle x^{2n}(t)\rangle = \int_{-\infty}^{\infty} x^{2n}\rho(x,t)\,dx. \tag{C.1}$$

Taking into account Eqs. (B.5) and (B.6), Eq. (C.1) takes the following form:

$$\langle x^{2n}(t)\rangle = \sum_{k=1}^{\infty}\int_{-\infty}^{\infty} x^{2n}\rho_k(x,t)\,dx = \frac{(2n)!}{2^n n!}e^{-\omega t}\sum_{k=1}^{\infty}\omega^{k-1}\int \mathcal{D}t_{k-1}\left[\Delta_k^2(t,\mathbf{t}_{k-1})\right]^n. \tag{C.2}$$

In this scenario, in order to extract information we must able to solve two problems: first we must be able to determine the general function $\mathcal{G}_{n,k}(t)$, which is the resulting expression after we perform all the integrals over Δ_k^{2n}, i.e.,

$$\mathcal{G}_{n,k}(t) \equiv \int \mathcal{D}t_{k-1}\left[\Delta_k^2(t,\mathbf{t}_{k-1})\right]^n;$$

and second, we must be able rewrite the summation $\sum_k \mathcal{G}_{n,k}$ into well known mathematical functions $\mathcal{G}_n(t)$, which would be the ones who give proper mathematical description. Probably a complete solution is not possible, at least not with this approach, but we're going to show that at least the mean squared displacement $\langle x^2(t)\rangle$ is computable.

First we need to realize that whenever we fix the value of n in Eq. (C.2), it is always possible (in principle) to compute the integrals of the variance $\Delta_k^2(t,\mathbf{t}_{k-1})$ for every value of k, because its defining Eq. (B.2) is composed of polynomials in $\mathbf{t}_{k-1}$ and exponentials of the form $e^{-\gamma t_m}$, both functional forms that always admit analytical representation for any integral in the domain of $\int \mathcal{D}t_{k-1}$. Secondly, the only possible result one can obtain after performing all integrations are functional forms that may be expressed in terms of polynomials in t and exponentials of the form $e^{-m\gamma t}$, where m assume all integer values in the interval $[0,n]$. Now, if we focus on a general expression for the mean square displacement, we may use these properties to write $\langle x^2(t)\rangle$ as a sum of three contributions, as in

$$\langle x^2(t)\rangle = e^{-\omega t}\sum_{k=1}^{\infty}\omega^{k-1}\mathcal{G}_{1,k}(t) = \sigma^2 e^{-\omega t}\left[e^{-2\gamma t}\sum_{k=1}^{\infty}\mathcal{P}_k^{(\mathrm{I})}(t) + e^{-\gamma t}\sum_{k=1}^{\infty}\mathcal{P}_k^{(\mathrm{II})}(t) + \sum_{k=1}^{\infty}\mathcal{P}_k^{(\mathrm{III})}(t)\right], \tag{C.3}$$

where $\mathcal{P}_k^{(\bullet)}(t)$ are polynomials in t. Since we're only able to compute the integrals for fixed values of k, the only way to determine the general expression of the polynomials is to use the result obtained from the first few values of k to infer their general behavior. If we start by computing the first polynomial, then the resulting expressions are all described by the general equation

$$\mathcal{P}_k^{(\mathrm{I})}(t) = \frac{\omega^{k-1}}{\gamma^{k+3}}\sum_{n=0}^{k-1}\frac{(\gamma t)^n}{n!}. \tag{C.4}$$

A more suitable expression is obtained when we consider some different representations of the incomplete Gamma function, as in

$$\Gamma(k,x) = e^{-x}(k-1)!\sum_{n=0}^{k-1}\frac{x^n}{n!} = \int_x^{\infty} z^{k-1}e^{-z}dz, \tag{C.5}$$

because now we may perform the summation over the index k to obtain an integral representation of this contribution:

$$\sum_{k=1}^{\infty}\mathcal{P}_k^{(\mathrm{I})}(t) = \frac{e^{\gamma t}}{\gamma^4}\int_{\gamma t}^{\infty} dz e^{-z}\sum_{k=1}^{\infty}\frac{(\omega z/\gamma)^{k-1}}{(k-1)!} = \frac{e^{\gamma t}}{\gamma^4}\int_{\gamma t}^{\infty} dz e^{z\left(\frac{\omega}{\gamma}-1\right)}. \tag{C.6}$$

It is easy to notice that this integral is only convergent if $\gamma > \omega$, but for now let's assume that this condition is always satisfied so we can get rid of any potential divergences. After solving the integral in Eq. (C.6), we may write this term's contribution to the MSD as

$$e^{-(2\gamma+\omega)t}\sum_{k=1}^{\infty}\mathcal{P}_k^{(\mathrm{I})}(t) = \frac{e^{-2\gamma t}}{\gamma^3(\gamma-\omega)}. \tag{C.7}$$

For the second and third polynomials we've obtained the inferred expressions

$$\mathcal{P}_k^{(\mathrm{II})}(t) = \frac{2}{\gamma^3\omega}\left[\frac{(\omega t)^k}{k!} - \left(\frac{\omega}{\gamma}\right)^k\sum_{n=0}^{k-1}\frac{(\gamma t)^n}{n!} - \left(\frac{\omega}{\gamma}\right)^k\delta_{k,\mathrm{even}}\right], \tag{C.8a}$$

$$\mathcal{P}_k^{(\mathrm{III})}(t) = \frac{2}{(\gamma\omega)^2}\frac{k(\omega t)^{k+1}}{(k+1)!} + \frac{2}{\gamma^3\omega}\left[\left(\frac{\omega}{\gamma}\right)^k - \frac{(\omega t)^k}{k!} + \left(\frac{\omega}{\gamma}\right)^k\left(\frac{1}{2}-\delta_{k,\mathrm{odd}}\right)\sum_{n=0}^{k-1}\frac{(-\gamma t)^n}{n!}\right]. \tag{C.8b}$$

To perform the summations we just need to know the power series representation of some regular functions, and for the cases shown in the previous equation we are going to use the following expansions:

$$\sum_{k=1}^{\infty}\left(\frac{\omega}{\gamma}\right)^k = \frac{\omega}{\gamma-\omega}, \tag{C.9a}$$

$$\sum_{k=1}^{\infty}\left(\frac{\omega}{\gamma}\right)^k\delta_{k,\mathrm{even}} = \left(\frac{\omega}{\gamma}\right)^2\sum_{k=0}^{\infty}\left(\frac{\omega}{\gamma}\right)^{2k} = \frac{\omega^2}{\gamma^2-\omega^2}, \tag{C.9b}$$

$$\sum_{k=1}^{\infty}\frac{(\omega t)^k}{k!} = e^{\omega t}-1, \tag{C.9c}$$

$$\sum_{k=1}^{\infty}\frac{k(\omega t)^{k+1}}{(k+1)!} = 1+e^{\omega t}(\omega t-1), \tag{C.9d}$$

$$\sum_{k=1}^{\infty}\left(\frac{\omega}{\gamma}\right)^k\sum_{n=0}^{k-1}\frac{(-\gamma t)^n}{n!} = \frac{\omega}{\gamma-\omega}e^{-\omega t}, \tag{C.9e}$$

$$\sum_{k=1}^{\infty}\left(\frac{\omega}{\gamma}\right)^k\sum_{n=0}^{k-1}\frac{(-\gamma t)^n}{n!}\delta_{k,\mathrm{odd}} = \frac{\omega}{\gamma}e^{-\gamma t}\int_{-\gamma t}^{\infty}dz e^{-z}\sum_{k=0}^{\infty}\frac{(\omega z/\gamma)^{2k}}{(2k)!} = \frac{\omega}{2}\left(\frac{e^{-\omega t}}{\gamma-\omega}+\frac{e^{\omega t}}{\gamma+\omega}\right). \tag{C.9f}$$

As a result, we may write the contributions from these polynomials as

$$e^{-(\gamma+\omega)t}\sum_{k=1}^{\infty}\mathcal{P}_k^{(\mathrm{II})}(t) = \frac{2}{\gamma\omega}e^{-\gamma t}\left[\frac{\gamma-2\omega}{\gamma^2(\gamma-\omega)} - \frac{1}{\gamma^2-\omega^2}e^{-\omega t}\right], \tag{C.10a}$$

$$e^{-\omega t}\sum_{k=1}^{\infty}\mathcal{P}_k^{(\mathrm{III})}(t) = \frac{2}{\gamma^2\omega}t - \frac{2(\gamma+\omega)^2+\omega^2}{\gamma^3\omega^2(\gamma+\omega)} + \frac{2}{\gamma\omega^2(\gamma-\omega)}e^{-\omega t}, \tag{C.10b}$$

and when we combine it together with Eq. (C.7) we obtain an analytical expression for the mean squared displacement

$$\langle x^2(t)\rangle = \frac{2\sigma^2}{\gamma}\left\{\frac{1}{\gamma\omega}t - \frac{2(\gamma+\omega)^2+\omega^2}{2(\gamma\omega)^2(\gamma+\omega)} + \frac{1}{\gamma-\omega}\left[\frac{e^{-2\gamma t}}{2\gamma^2} + \frac{e^{-\gamma t}}{\omega}\left(\frac{\gamma-2\omega}{\gamma^2} - \frac{e^{-\omega t}}{\gamma+\omega}\right) + \frac{e^{-\omega t}}{\omega^2}\right]\right\}. \tag{C.11}$$

After introducing the rescaled parameters defined in Eq. (14), the resulting expression will be the one used in the main text, i.e., Eq. (13).

D Extension to other probability distributions

The linear combination of accelerations given by Eq. (11) in the main text may be written as

$$x_k = \sum_{n=1}^{k^*} \frac{a_n}{\gamma^2}\left[\gamma t_n + \left(e^{-\gamma t_n} - 1\right)\exp\left(-\gamma \sum_{j=n+1}^{k^*} t_j\right)\right] = \sum_{n=1}^{k^*} c_n\left(t, \mathbf{t}_{k-1}\right) a_n. \tag{D.1}$$

Now, instead of considering the accelerations to be normally distributed, let us assume a general representation with the only requirement that it satisfies $\langle a\rangle = 0$. In the same way as we wrote a conditional probability for the Gaussian process in Eq. (B.1), we can write a 'conditional' characteristic function for the random variable x_k in Eq. (D.1) as

$$\Phi_k\left(z, t, \mathbf{t}_{k-1}\right) = \prod_{n=1}^{k^*} \mathit{\Phi}_a\left(c_n z\right), \tag{D.2}$$

where $\mathit{\Phi}_a(z)$ is the characteristic function of the random variable a. The characteristic function of the whole process is obtained by summing over all possible bouts (as regard to the number and durations), just as we did in Eqs. (B.3-B.5), which leads to

$$\Phi(z, t) = e^{-\omega t} \sum_{k=1}^{\infty} \omega^{k-1} \int \mathcal{D} t_{k-1} \Phi_k\left(z, t, \mathbf{t}_{k-1}\right). \tag{D.3}$$

Now let us assume that the characteristic function $\mathit{\Phi}_a(z)$ is at least twice differentiable at the point $z = 0$, which means that the second moment of the distribution exists. If ν_a^2 is the variance of the probability distribution function $\phi(a)$, then Eq. (D.2) can be rewritten as

$$\Phi_k\left(z, t, \mathbf{t}_{k-1}\right) = 1 - \left(\frac{\nu_a^2}{2}\sum_{n=1}^{k^*} c_n^2\right) z^2 + O\left(z^3\right). \tag{D.4}$$

The MSD of the whole process is obtained from the characteristic function as follows:

$$\left\langle x^2(t)\right\rangle = -\left.\frac{d^2}{dz^2}\Phi(z, t)\right|_{z=0}, \tag{D.5}$$

Having inserted (D.4) into (D.3) and making use of (D.5), we obtain:

$$\left\langle x^2(t)\right\rangle = \nu_a^2 e^{-\omega t} \sum_{k=1}^{\infty} \omega^{k-1} \int \mathcal{D} t_{k-1} \sum_{n=1}^{k^*} c_n^2\left(t, \mathbf{t}_{k-1}\right). \tag{D.6}$$

Now if we compare $\nu_a^2 \sum_{n=1}^{k^*} c_n^2\left(t, \mathbf{t}_{k-1}\right)$ to the definition of $\Delta_k^2\left(t, \mathbf{t}_{k-1}\right)$ in Eq. (B.2), it is easy to see that they are essentially the same expressions subject to the substitution ν_a^2 instead of σ^2. Since the expression for the MSD in Eq. (C.3) is the same as in Eq. (D.6), we conclude that, subject to the replacement of σ^2 by ν_a^2, the expression (C.11) is valid for any probability distribution with a finite second moment.
